# Supplementary material for: Validation of robust radiobiological optimization algorithms based on the mixed beam model for intensity-modulated carbon-ion therapy
Source: PLoS One. 2023 Jul 28;18(7):e0288545. doi: 10.1371/journal.pone.0288545 (PMC10381094; doi:10.1371/journal.pone.0288545)
Supplement: Attachment — Submitted filename: Response to Reviewers_v0.1.docx [file pone.0288545.s001.docx]

Please note that line number in **Answer** is the number in the Manuscript (i.e. manuscript without track changes).

Additional Editor Comments (if provided):

This manuscript evaluates some key features of a TPS in supporting IMCT. These include both the accuracy of physical and biologic dose calculation, plus the abilities of robust optimization. There are some valuable results such as the measurement of survival factions for in comparison to calculation. However, the manuscript is very difficult to read in current form. Overall, the manuscript is not written in a clear, consistent and coherency way. There are a lot of guessing on what the author is expressing. I strongly suggest the authors rewrite the manuscript with professional English editing.

**Answer** Thank you for your comment. We requested a professional English editing twice before submission. We hope this meets the criteria.

Some specific comments include:

• It is unclear on the purpose of the manuscript. Most of work described in the manuscript is about validation of the TPS. Not much on the development except the description of SC and WC algorithms in the method and material section.

**Answer** Thank you for your indication. In the present study, the computational efficacy of the newly developed IMCT algorithms was analyzed for the first time based on the mixed beam model with respect to the physical and biological doses; moreover, the validity and effectiveness of the robust radiobiological optimization were verified. In the method and material section, we focused to write carbon-ion therapy specific development for spot control (SC) and worst case (WC) algorithms, especially in terms of biological dose calculation. The radiobiological optimization which uses biological effect instead of physical dose is newly developed for SC and WC optimization algorithms. In addition, the SC algorithm is modified in this study as describing in line194-199: the risk index was simply calculated using geometrical information in the algorithm created by our group instead of dose calculation derived alternatively in a prior study.

• Physical dose calculation validated by in house software. What makes the in house software the standard?

**Answer** Thank you for pointing this out. The dose calculation algorithm in commercially available software is unclear that how the software implements the dose calculation algorithm though the dose calculation algorithm itself is open for the users. The in-house software is created by the same dose calculation algorithm to see whether or not a big difference is not shown between the commercially available and in-house software. Because we know how the dose calculation algorithm is implemented, the in-house software could be the standard.

5. Review Comments to the Author Please use the space provided to explain your answers to the questions above. You may also include additional comments for the author, including concerns about dual publication, research ethics, or publication ethics. (Please upload your review as an attachment if it exceeds 20,000 characters)

Reviewer #1:

The authors applied the mixed beam model in the physical and radiobiological dose optimization for intensity modulated carbon therapy (IMCT). Besides TPS, they have developed an independent dose calculation engine based on Python. Most importantly, they conducted cell irradiation to validated the accuracy of the TPS and independent dose engine. They found that the robust radiobiological optimization enhanced the sensitivity of the examined error by up to 19% compared to the conventional IMCT. Using the worst case scenario algorithm can even enhance the robustness more than the spot control algorithm. This is a very comprehensive study including TPS, independent dose verification engine, and cell irradiation experiments, phantom study, and optimization in patient case. I would recommend its acceptance for publication after minor revision. Nevertheless, I have to admit that this paper is really long and not easy to be understood especially for those who do not have experience in carbon therapy. Some comments are listed below:

(1) Page 8, line 37, it should be “surviving fraction”, not “survival fraction”. Please also change the label in figure 4.

**Answer** Thank you for highlighting this out. We changed the words, “survival fraction” to “surviving fraction” in the text and figure 4.

(2) Page 8, line 47, what is “-a 0.6%”?

**Answer** Thank you for pointing this out. It is “a −0.6%”. We modified it.

(3) page 16, line 279, in each plate. What type of cell culture plate did you use? Single-well petri dish?

**Answer** Thank you for your comment. We seeded the cells to 6 cm φ cell culture dishes (Corning, Somerville, MA, US). So, we added the product information and rewrote our manuscript as below (line279-284).

After carbon-ion irradiation, 500 or 1000 cells were seeded in 6 cm φ cell culture dishes (Corning, Somerville, MA, US), with three dishes per one condition. These dishes were incubated for 2 weeks. Then, the cells were fixed with formaldehyde (10% solution, Sigma Aldrich) for 20 min before staining with crystal violet (FUJIFILM Wako Pure Chemical Corporation, Osaka, Japan). The dishes were then washed with water. When a cell population reached a size of ≥50 cells, it was considered a colony [29].

(4) page 31, the last paragraph regarding the discussion of LET painting. I don’t think it is necessary to mention this because in carbon ion therapy, LET is no longer an appropriate quantity in plan optimization and evaluation. You have conducted the robustness analysis of radiobiological dose, which is the appropriate quantity. Because multiple secondary charged particles are generated in carbon therapy, the appropriate physical quantity is lineal energy and/or specific energy in microdosimetry, rather than LET.

**Answer** Thank you for your comment. The LET is a macroscopic quantity as you pointed out. Using dose-averaged LET is clinically acceptable at least in carbon-ion therapy though lineal energy and/or specific energy is more accurate to describe beam quality. In particle therapy, beam quality based optimization is of interest because it is begun to prove that focusing high energy spectrum is effectiveness on hypoxic tumors (Inaniwa et al., Phys Med Biol. 2017 Jun 21;62(12):5180-5197). We added the following sentence in the text (line 665-668).

The dose-averaged linear-energy transfer (LET) is clinically used in carbon-ion therapy [9] though there are other physical quantities to describe beam quality such as lineal energy and/or specific energy which are distinguished based on whether or not considering stochastic nature of the carbon-ion beam [33].

(5) page 39, figure 4, there are results from experiment, experiment with SFUD and independent calculation, why isn’t there the surviving fraction predicted in your TPS by WC or SC algorithms?

**Answer** Thank you for your question. The TPS (here VQA) does not have a function to show the value of surviving fraction to users. The surviving fraction predicted in VQA by WC or SC algorithms is the same as one predicted in the independent calculation which implements the same dose calculation algorithm as the commercially available VQA.

Reviewer #2:

This manuscript discusses an investigation of robust optimization strategies for intensity modulated carbon ion therapy (IMCT). The study included both physical uncertainties and biological doses using two optimization methods, spot control and worst case for testing phantom and patient cases. It also included both calculated biological effect and survival rate validation of a human salivary gland cell line. Both plan robustness and biological effect are important topics in clinical particle therapy currently and thorough investigations are needed. I think this manuscript should be of great interest for particle therapy researchers and practitioners. The authors did a good job in explaining the methodologies and providing detailed supporting data. However, I have several comments as summarized in below with an aim towards improving the clarity of the manuscript. I would suggest the authors to consider revising the title of this study, as well as abstract and other discussions if related. First, it seems the robust biological optimization algorithms had been developed in previous studies or from existing TPS, e.g., spot control (SC) and worst case (WC) optimization algorithms, and RBE optimization based on local effect or MK models. I think “development” might not be appropriate in the title. Some options could be “evaluation” or “assessment”, etc. Secondly, the term “mixed beam” is not descriptive. It could mean a mix of photon and particle beams, or a mix of different beam lines, or different RBE models, without a definition. It should be stated clearly what “mixed” means in the title and its first appearance in the main text.

**Answer** Thank you for your suggestions. About the first question, radiobiological optimization which uses biological effect instead of physical dose is newly developed for spot control (SC) and worst case (WC) optimization algorithms. In addition, the SC algorithm is modified in this study as describing in line194-199: the risk index was simply calculated using geometrical information in the algorithm created by our group instead of dose calculation derived alternatively in a prior study [21]. About the second question, the mixed beam model is general term in the context of RBE especially in carbon-ion therapy as well as the local effect or MK models. However, we added the explanation as follows (line88-89).

Here the “mixed” means a mixed linear-energy transfer (LET).

The measurement of survival factions for the selected cell line presented interesting results for readers. However, it was not discussed with more detail. It would be more helpful if the authors could comment on some important aspects, such as the correlation between calculated and measured results, any insights from the experiments, the progress concerning previous validation, the current approach, and maybe future works.

**Answer** Thank you for your comments. In our experiment condition with HSGc-C5 cells, the surviving fraction is systematically about +10% higher than that of calculation which is expected as 10%. The discrepancy was also reported in our previous paper (Fujitaka et al., Journal of Applied Clinical Medical Physics. 2021;22(7):77-92). It was reported that the small discrepancy between the computed outcomes and measurements was potentially attributed to a slight modification in the sensitivity of the used HSG cells (line614-617). We added the following sentence (line617-619).

The systemic discrepancy itself is not problematic as long as the similar difference is observed because of the slight modification in the sensitivity of the cell. A similar difference was also reported from the same research group[6].

Some specific comments include:

• Line 54. It could be more appropriate to state “the impact of range and position uncertainties”, because robust optimization does not reduce uncertainties directly.

**Answer** Thank you for pointing this out. We modified the sentence as you suggested (line53).

The robust radiobiological optimizations lowered the impact of range and position uncertainties considerably in the examined scenarios.

• Line 134. It may be not necessary to add “independently from non-IMCT” as it is inherent in IMCT that each field is independently modulated. Same for Line 148.

**Answer** Thank you for highlighting this out. The words are deleted from the text.

• Line 152. How are alpha_ij and beta_ij determined? Details or a previous publication could be useful.

**Answer** Thank you for your comment. We added the following sentence with the reference describing how to determine alpha_ij and beta_ij (line153).

These LQM parameters are functions of the LET and are obtained from [6].

• Line 220. Is there a typo in equation (16)? Terms in the bracket seem identical.

**Answer** Thank you for your question. The equation is correct. The first term is the function of $\theta$ and the variable is $\max_{s} e_{i}^{s}-e_{max}$.

• Line 233. Is the “dose calculation engine” based on analytical model or Monte Carlo? Is there any validation work for it, maybe in another work?

**Answer** Thank you for your comment. It is based on an analytical model. The implemented algorithm was validated with a study (Yagi et al., PLoS ONE 17(5): e0268087. 2022).
