# Supplementary material for: Validation of robust radiobiological optimization algorithms based on the mixed beam model for intensity-modulated carbon-ion therapy
Source: PLoS One. 2023 Jul 28;18(7):e0288545. doi: 10.1371/journal.pone.0288545 (PMC10381094; doi:10.1371/journal.pone.0288545)
Supplement: Attachment — Submitted filename: Response to Reviewers_R2_v0.0.docx [file pone.0288545.s002.docx]

Please note that line number in **Answer** is the number in the Manuscript (i.e. manuscript without track changes).

Additional Editor Comments:

This manuscript evaluates some key features of a TPS in supporting IMCT. These include both the accuracy of physical and biologic dose calculation, plus the abilities of robust optimization. The study provides some valuable results in the advancement of IMCT and is clearly worth the publication. Both reviewers feel the revision addresses their previous comments and concerns. I agree the revision has significant improvement from previous submission. However, there are still some concerns as listed below:

1. The revision is more readable. The abstract and introduction sections can be improved to make the manuscript appear in a clear, consistent and coherency way.

**Answer** Thank you for your comment. We requested professional English editing which includes analyzing the development of the argument and the appropriateness of the content twice before submission. We hope this meets the criteria of making the manuscript appear in a clear, consistent and coherency way.

2. Strong recommendation on changing the title. The manuscript focused on the validation of the dose calculation of robust optimization algorithms. We expressed concern on using the world “development” which mismatch the purpose of the study.

**Answer** Thank you for your suggestion. The title was changed to “Validation of …” instead of “Development of…”.

3. Physical dose calculation validated by in house software. What makes the in house software the standard?

**Answer** Thank you for pointing this out. One validation method is comparing the physical dose calculation in the new TPS with the physical dose calculation in a validated software that implements the same physical dose calculation algorithm as the new TPS. The physical dose calculation algorithm in the TPS has been validated with the measurements in the previous study and standardized [1]. The physical dose calculation algorithm implemented in the in-house software is the same as the validated TPS. In this study, based on the validated physical dose calculation algorithm, the dose calculation algorithm for IMCT was evaluated. The dose calculation algorithm in commercially available software is unclear that how the software implements the dose calculation algorithm, especially for IMCT though the dose calculation algorithm itself is open to the users. The in-house software is created by the same dose calculation algorithm as the TPS according to the opened algorithm to see whether or not a big difference is not shown between the commercially available and in-house software. Because we know how the dose calculation algorithm is implemented and the basic physical dose calculation algorithm was validated in the previous study, the in-house software can be the standard.

1.	Yagi M, Tsubouchi T, Hamatani N, Takashina M, Maruo H, Fujitaka S, et al. Commissioning a newly developed treatment planning system, VQA Plan, for fast-raster scanning of carbon-ion beams. PloS one. 2022;17(5):e0268087.
